# An overview of the neuro-cognitive processes involved in the encoding, consolidation, and retrieval of true and false memories

**DOI:** 10.1186/1744-9081-8-35

**Published:** 2012-07-24

**Authors:** Benjamin Straube

**Affiliations:** 1 Department of Psychiatry and Psychotherapy, Philipps-University Marburg, Marburg, Germany

**Keywords:** False memory, Memory biases, Brain, Functional magnetic resonance imaging, Self-reference, Encoding, Consolidation, Retrieval

## Abstract

Perception and memory are imperfect reconstructions of reality. These reconstructions are prone to be influenced by several factors, which may result in false memories. A false memory is the recollection of an event, or details of an episode, that did not actually occur. Memory formation comprises at least three different sub-processes: encoding, consolidation and the retrieval of the learned material. All of these sub-processes are vulnerable for specific errors and consequently may result in false memories. Whereas, processes like imagery, self-referential encoding or spreading activation can lead to the formation of false memories at encoding, semantic generalization during sleep and updating processes due to misleading post event information, in particular, are relevant at the consolidation stage. Finally at the retrieval stage, monitoring processes, which are assumed to be essential to reject false memories, are of specific importance. Different neuro-cognitive processes have been linked to the formation of true and false memories. Most consistently the medial temporal lobe and the medial and lateral prefrontal cortex have been reported with regard to the formation of true and false memories. Despite the fact that all phases entailing memory formation, consolidation of stored information and retrieval processes, are relevant for the forming of false memories, most studies focused on either memory encoding or retrieval. Thus, future studies should try to integrate data from all phases to give a more comprehensive view on systematic memory distortions. An initial outline is developed within this review to connect the different memory stages and research strategies.

## Introduction

Just like perception, memory is a constructive process that is sometimes prone to error and distortion (e.g., [1-3]). Various memory systems can be distinguished in terms of the different kinds of information they process and the principles by which they operate [4-6]. Consequently those systems are differentially prone to false memories [5,7]. Declarative memory is a long term human memory system that refers to memories which can be consciously recalled such as facts, knowledge or events. An important principle for the declarative memory is the ability to detect, encode and retrieve what is unique about a single event occurring at a particular time and place. Declarative memory is highly flexible, representational and provides a way to model the external world. Since declarative memories are (due to their flexibility) especially prone to errors and distortions, the current review focuses specifically on the declarative memory system. Declarative memory can be further divided into semantic memory (facts about the world) and episodic memory (the capacity to re-experience an event in the context in which it originally occurred) [7,8]. It has been shown that episodic memory requires the participation of brain systems in addition to those that support semantic memory, especially the frontal lobes [8-11]. With regard to false memories, it has been assumed that the semantic memory system is less vulnerable to involuntary transformations and distortions than the episodic memory system [7]. In line with this assumption the evidence about false memories particularly refers to episodic or “episodic like” aspects of memories, which often requires that at least two aspects of the original encoding situation be remembered (e.g., sentence content and body orientation [12]).

Since episodic memory is flexible, representational and often contains multiple pieces of information from the encoding situation, it comprises an especially constructive process that is prone to error and distortion (e.g., [1-3]). The theory that false memories are a byproduct of a constructive memory system is supported by a considerable number of behavioral and neuroimaging studies (for a review see [13]). It has been demonstrated that human memory is influenced by several factors, such as prior knowledge (e.g., [11,14,15]), current mental state or emotions (e.g., [11,13,14,16-20]), and therefore does not reflect a perfect documentation of the external world. Thus, what is retrieved from memory can significantly differ from what was initially encoded (e.g., [12,13,21]). The most intriguing are false memories of specific psychiatric patients (e.g., those presenting confabulations [22-24]) and the most disastrous are those leading to false decisions in juristic context [25-27]. Specific examples about the formation of false memories will be given in this review article to illustrate the variety of memory errors related to diverse stages of processing. Different sources of false memories at encoding, consolidation and retrieval will be discussed and compared. This article will finish with a research agenda for the comprehensive investigation of the formation of false memories.

### Encoding

Processes like imagery (e.g., [21,28-30]), self-referential processing (e.g., [12]) or spreading activation (e.g., [31-35]) can lead to the formation of false memories, already at encoding.

An example for the formation of false memories at encoding is seen in errors that occur when a memory of an imagined event is later falsely remembered as a memory of a perceived event (e.g., [21,28-30]). These errors may arise as a consequence of similarities between how imagined and perceived events are encoded, as well as similarities among the event features that are reactivated during retrieval [36,37]. It has been suggested, that such confusions may serve as a basic mechanism for the induction of many types of false memories [21,28-30]. It has further been hypothesized that brain networks during encoding are related to subsequent confusion of imagined and perceived events [28]. Accordingly, activations in the precuneus and inferior parietal regions in response to words were found during the encoding stage to be greater when participants subsequently claimed to have seen the corresponding object than when they did not produce a subsequent false memory [28]. These results suggest that brain activity of the precuneus - which might indicate the engagement of visual imagery - during encoding can lead to falsely remembering something that was only imagined. Thus, visual imagery or everything like ‘fantasy’ can influence memories directly during encoding by providing a biased or mentally changed episode for the episodic memory system, which is likely to be later remembered in a similarly biased or changed way.

Beside imagery, there are two further theoretical accounts of false memory encoding (e.g., [35]): The ‘spreading activation theory’ (e.g., [31-35]) and the ‘fuzzy trace view’ (e.g., [35,38-41]). These views were discussed particularly with regard to findings of the Deese–Roediger–McDermott (DRM) paradigm, in which subjects were presented with a list of semantically related words (e.g., bed, rest, wake, tired, dream, etc.) that converge on a single, non-presented critical item such as sleep [42,43]. In line with the ‘spreading activation theory’ (e.g., [31-35]), it is assumed, that activation flows from list items to critical lures in the semantic network. Consequently, the associative activation of related or similar items is stored and subsequently misattributed to actual encounters. According to the ‘fuzzy trace view’ (e.g., [35,38-41]), studying a list of related words or pictures leads to formation of ‘verbatim traces,’ which contain item-specific information, as well as ‘gist traces,’ containing the general topic of the list. Both theories rely on the assumption that the formation of false memory traces (spreading activation, gist traces) may be a byproduct of perceptual and semantic elaboration processes [35].

In a recent functional magnetic imaging (fMRI) study, an adapted version of the DRM-paradigm was used to investigate the neural activity associated with the formation (encoding) of true versus false memories [35]. The materials were 72 categorical 6-word lists, each consisting of the 6 most typical instances (e.g., cow, pig, horse, chicken, sheep, goat) of a natural/artificial category (e.g., farm animal). In the test phase, True-word, False-word, and New-word trials were presented and subjects responded by pressing one of four keys according to whether the word was judged to be ‘definitely old,’ ‘unsure if old,’ ‘unsure if new,’ or ‘definitely new.’ In their data analyses the authors investigated, which brain regions show true memory formation activity (i.e., positive covariation between encoding trial activations and later hit rate for words from those trials) and which brain regions show false memory formation activity (i.e., positive covariation between encoding trial activations and later false alarm rate for semantic associates of words from those trials).

They found that activity in the left ventrolateral prefrontal cortex (PFC) and visual areas at encoding contribute to both true (old item) and false memories (e.g., new item of the old category). By contrast, activity in a left posterior medial temporal lobe (MTL) and early visual areas at encoding contributes mainly to the formation of true memories. This finding supports the view that false memories are due, at least in part, to processes that take place at the time of encoding, such as the spreading activation view (e.g., [31,33,34,44-46]) and the fuzzy trace view (e.g., [38-41]). The authors concluded that the formation of false memories is largely an unintended consequence, or byproduct, of elaborative semantic and visual encoding processes [35].

The fMRI findings suggest that attention or executive processes related to the PFC might be relevant for both true and false memories, whereas memory processes of the left posterior MTL are specific for true memories. However, PFC activations have also been observed with regard to the effect of schemas on memory encoding and retrieval [47,48] and seem to play an specific role in remote memory processes [49]. Thus, PFC activation in context of the DRM-paradigm at encoding can also reflect such schema based memory processes instead of pure executive or attention processes.

In a proprietary study we aimed to determine the neural correlates of self-referential encoding processes induced by scenes of egocentric vs. allocentric social interactions [12]. During fMRI data acquisition, participants were presented with video clips of an actor speaking and gesturing directly towards them (egocentric) or towards an unseen third person (allocentric). After scanning, recognition performance for sentences (items) and communication context (source) was gathered. We found no differences between the recognition of sentences spoken in egocentric and allocentric contexts. However, when asked about the communication context (‘Had the actor directly spoken to you?’) participants tended to believe falsely that the actor had directly spoken to them during allocentric conditions. Greater activity in the hippocampus was related to correct context memory, whereas the ventral anterior cingulate cortex was activated for subsequent inaccurate context memory. We observed increased activation in the bilateral and medial frontal cortex, the basal ganglia and the left parietal and temporal lobe for the interaction between encoding context and context memory. These data indicate that memories of social interactions are prone to be remembered egocentrically. Self-referential encoding processes reflected in increased frontal activation and decreased hippocampal activation might be the basis of correct item memory but false context memory of social interactions.

Various medial cortical regions have been found to be active during self-referential processing, including the medial orbital prefrontal cortex (MOFC), the ventromedial prefrontal cortex (VMPFC), the sub/pre- and supragenual anterior cingulate cortex (PACC, SACC), the dorsomedial prefrontal cortex (DMPFC), the medial parietal cortex, the posterior cingulate cortex, the retrosplenial cortex and precuneus [12,50-54]. These regions have been subsumed under the term “cortical midline structures” (CMS) and speculatively characterized as an anatomical and functional unit related to the self [55]. Our study is an example of how self-referential processes probably located in the cortical midline structures, and, specifically, the ventral anterior cingulate cortex, can lead to the formation of false memories by influencing encoding processes.

Thus processes like imagery, self-reference or spreading activation can lead to the formation of false memories at encoding. Data from neuroimaging studies suggest that perception (e.g., in occipital and parietal brain structures [28]) and storage processes (e.g., hippocampus activation for true vs. false memories [35] and hippocampus deactivation for false context memories [12]) are relevant during encoding for the creation of a false memory representation. Those biases in memory encoding can be elicited through imagery (e.g., [28]), spreading activation or the construction of a gist trace (e.g., [35]) as well as self-referential processes (e.g., [12]). The frontal lobe seems to play a key role in influencing those processes usually involved in the formation of true memories.

### Consolidation

Information stored in memory is also prone to distortions due to new incoming information (e.g., ‘retroactive interference’ [56,57]) and sleep or sleep deprivation [58-62].

Specifically, sleep plays an active role in memory consolidation [60,63,64]. Whereas previous investigations suggested an effect of sleep especially for non-declarative memory performances (see [65]) newer studies provide evidence for sleep on declarative memory [66-68] and declarative memory distortions [58-62], as well.

During sleep, newly acquired memory traces are not only strengthened in distinct neural circuits, but additional new memory traces are also established for long-term storage and integrated within pre-existing long-term memories (“consolidation”, see [65,69]). This active reorganization can also lead to the formation of false memories, as the memory representation can differ from what was originally encoded after active restructuring and integration within pre-existing representations [70,71]. Thus, false memories can be created during consolidation as new and stable knowledge representations, which do not reflect the original encoded material but generalize it to semantically associated knowledge. By this way, sleep itself may promote false memories during memory consolidation [60].

For example Diekelmann and colleagues investigated whether sleep after learning and sleep deprivation at retrieval enhance the generation of false memories in a free recall test [60]. According to the DRM false memory paradigm, subjects learned lists of semantically associated words, lacking the strongest common associate or theme word. Free recall was tested after 9 h following a night of sleep, a night of wakefulness (sleep deprivation) or daytime wakefulness. Compared with memory performance after a retention period of daytime wakefulness, both post-learning nocturnal sleep, as well as acute sleep deprivation at retrieval significantly enhanced false recall of theme words in subjects with low general memory performance. These data suggest two different ways in which sleep affects the formation of false memories through semantic generalization. The first influences the consolidation of a memory trace per se, probably by active reorganization of the trace in the post-learning sleep period. The second is related to the recovery function of sleep and affects cognitive control processes of retrieval (see retrieval, below). The finding that sleep effects were confined to subjects with generally low memory performance fits well with previous studies indicating that the effect of sleep on memory consolidation is greater for weak than strong memory traces (e.g., [72,73]). In high performing subjects memory traces might be especially strong and not as vulnerable to consolidation specific distortions as in low performing subjects.

A recent fMRI study investigated the influence of sleep and lack of sleep on the cerebral correlates of accurate and false recollections [62]. After encoding lists of semantically related word associates, half of the participants were allowed to sleep, whereas the others were totally sleep deprived on the first post-encoding night. During a subsequent retest fMRI session taking place 3 days later, participants made recognition memory judgments about the previously studied associates, critical theme words, and new words unrelated to the studied items. Sleep in contrast to sleep deprivation, enhanced accurate and false recollections. No significant difference was observed in brain responses to false or illusory recollection between sleep and sleep deprivation conditions. However, accurate and false recollections were both associated with responses in the hippocampus and retrosplenial cortex solely after sleep. The authors suggest, that these data indicate that sleep does not selectively enhance false memories, but rather tends to promote general consolidation in hippocampo-neocortical circuits for true and false memories [62]. They further observed that during encoding, hippocampal responses were selectively larger for items subsequently accurately retrieved than for material leading to illusory memories. Thus the early organization of memory during encoding seems to be a major factor influencing subsequent production of accurate or false memories.

Sleep is an important factor for the creation of true and false memories during consolidation. However, as the studies of Diekelmann and Darsaud illustrate, it is often difficult - but important - to disentangle those consolidation processes from relevant encoding and retrieval processes.

In addition to sleep or sleep deprivation, new incoming information can lead to memory distortions and false memories (e.g., ‘retroactive interference’ [56,57]). Whereas proactive interference occurs when earlier memories influence new memories at encoding, retroactive interference takes place when old memories are changed by new incoming information at the consolidation stage. Thus, misleading post-event information (MPI) can produce changes of old memories [56,57]. In an famous experiment, subjects watched a film in which there were several car accidents [74]. Afterwards subjects were separated into three groups that each received different questions. While the control group was not asked about the speed of the cars, questions for the other groups were manipulated with regard to a specific key word. One group was asked about the speed when the cars hit each other, while in the other group the verb ‘smashed’ was used in the question. One week later all subjects were asked whether they saw broken glass in the films. Both the estimation of speed and the number of subjects that reported that they saw broken glass increased progressively from the control group to the third group [74]. This misinformation effect (e.g., [75-79]) demonstrates that suggestible and more detailed information receives after having made the actual memory can replace or transform the old memory. Recent fMRI studies investigated brain activity associated with the misinformation effect [78,80]. In one study, for example, subjects were shown a sequence of photographs [80]. Afterwards the same subjects listened to a narrative, which included misleading information about the pictures. Then, during fMRI data acquisition, they performed a memory test for the photographs. Similar patterns of brain activity were found for both true and false memories. However, the true memories showed somewhat more activation in the visual cortex (probably due to the visual information) while the false memories showed somewhat more activation in the auditory cortex (probably derived from the auditory narrative). These results are in line with the sensory reactivation hypothesis [81-83], which in part proposes that the same sensory regions activated in the brain during encoding will be reactivated during retrieval. These results suggest that there may be differing brain activation patterns for true and false memories when they are encoded in different sensory modalities.

In the other study [78], fMRI data were collected as participants studied eight vignettes (original event phase). After that, participants studied the same vignettes during scanning. However, this time changes to several details were included in the stories, serving as the misinformation (misinformation phase). Two days later, their memories for the original event were assessed. Activity that subsequently led to true and false memories was examined during both encoding phases. The researcher observed that an interaction of encoding processes in the MTL and PFC is critical for true and false memory formation in this paradigm, because activity during both encoding phases predicted whether the original event information or the inaccurate misinformation was reported. In the left hippocampus tail and perirhinal cortex, a predictive item-encoding pattern was observed. Thus, during the original event phase, activity was greater for true than false memories, whereas during the misinformation phase, activity was greater for false than true memories. By contrast, in other regions, especially in the superior frontal gyrus, a pattern suggestive of source encoding was observed, in which activity for false memories was greater during the original event phase than the misinformation phase. Together, these results suggest that specific item encoding (e.g., hippocampus) and source encoding (e.g., PFC) processes play a critical role in determining true and false memory outcome in misinformation paradigms. The authors concluded that components of the MTL are not selectively involved in one type of information processing. Their data suggested that different forms of encoding can occur even within the same structure of the MTL [78].

Together, the studies demonstrate how false memories can be created in the consolidation stage due to post-event information. Memory updating processes, related to perceptual recombination of recall cues together with new information mediated by memory and attention processes, are most likely involved in these effects of post event information. The imaging data support this assumption by demonstrating activation in perceptual brain regions [80], the MTL [78] and the PFC [78]. Thus, binding processes of the hippocampus (and later on the PFC) might be involved in binding together single elements of an episode (e.g. recall cues together with new information) stored across different parts of the brain [49].

The presented data illustrate how memories can be affected or distorted during consolidation due to new incoming information [56,57] and sleep or sleep deprivation [58-62]. Different mechanisms have been assumed to be relevant for those distortions. Whereas ‘retroactive interference’ due to new incoming information is most likely dependent on encoding and memory updating processes, sleep might affect the formation of false memories through semantic generalization by active reorganization of the memory trace in the post-learning sleep period.

### Retrieval

People sometimes believe they recognize things that they have never actually encountered, for example, confusing a stranger with an old acquaintance. False memory generation at retrieval is predominantly dependent on perceptual processes related to retrieval cues or tasks, the recall of information from memory and related executive functions (see below). With regard to the retrieval phase, false memories occur especially frequent under free recall conditions (e.g., [84-86]), but can also be observed in recognition experiments (e.g. [87,88]). However, since false memories can only be measured with tasks requiring some form of retrieval on behavioral level, it is difficult conclude that the production of false memories at retrieval are not due to encoding or consolidation processes (see above). Despite the fact that encoding and consolidation are potential candidates for the creation of false memories (as reported above), most studies focused on the retrieval processes leading to false responses in free or cued recall as well as recognition tasks.

Some processes that are important during retrieval are related to the retrieval cues when they provide false information as demonstrated with the misinformation paradigm. Effects of misinformation were already discussed in the consolidation section, since memories might be changed by suggestible retrieval cues even before the actual retrieval. Constructive or adaptive memory updating processes (see [89]), initiated by the processing of recall cues together with new information and related memory and attention processes are assumed to be involved in these effects of post event information. Perceptual brain regions [80], the MTLs [78] and the PFC [78], in particular, are involved in creating those memories.

Other aspects of false recognition were investigated with the DRM-paradigm ([43,90], see above). This paradigm is often used to investigate false memory at the retrieval phase, since subjects demonstrate a high rate of false memories for the critical item across a variety of experimental situations (see [13] for a review).

It is assumed that not only encoding and consolidation processes (see above), but retrieval mechanisms, as well, are affected by spreading activation across a wide variety of cognitive tasks (e.g., [34,45]). The assumption is that related concepts are linked in memory and that when one item or concept in memory is activated (via encoding or retrieval), the activation spreads to other related concepts [46]. The activation monitoring theory (e.g., [13,84,91]) is based on the idea that spreading activation works in combination with a more controlled, monitoring process at retrieval that allows subjects to make attributions about the source of the activation (e.g., [36]). Thus subjects may use information not only from activated concepts or stimuli at retrieval, but must also rely on a monitoring process to separate those activated items that were studied from those that were not studied. Here especially executive processes might play an important role in monitoring the retrieval processes and verify the accuracy of memories. In line with this assumption, results from sleep research indicate that a prolonged loss of sleep can enhance false memories [60,61] probably due to reduced source and reality monitoring [92,93].

False memories at retrieval, has been investigated extensively in both neuropsychological and neuroimaging research (for a review, see [2]). Several studies have compared the neural activity that accompanies genuine recognition of studied items and false recognition of novel items (e.g., [82,94,95]). Those neuroimaging studies have shown increased neural activity in regions of the medial and lateral frontal cortex during false recognition (e.g., [82,94,95]). Additionally, patients with frontal lobe damage have increased incidences of false recognition (e.g., [96-99]). These studies suggest that the frontal cortex plays an important role in monitoring processes related to false recognition.

However, neuroimaging studies of false recognition also reveal a remarkable overlap between the brain regions activated during true and false recognition (e.g., [2,95,100-102]). Thus, it is likely that there is also an overlap in the sub processes that contribute to true and false recognition [11].

It has been suggested that the only difference between true and false memories might be the degree of sensory perceptual information at recognition. This idea is supported by fMRI data showing greater activity for true recognitions than false recognitions in sensory brain regions (e.g., [94,95,100,101]). Together with findings of an involvement of the PFC during false recognitions it is assumed that true memories contain more perceptual and sensory information, whereas false memories contain more information about cognitive processes (e.g., [11]). The engagement of perceptual regions of the brain might reflect the retrieval of sensory information, which should be present especially for true memories (‘sensory reactivation hypothesis’, [2,11]).

In addition to successful retrieval of perceptual aspects of a memorized event, reconstructive processes combining information from different sources are also necessary under specific task conditions. Failures in such reconstructive processes are probably a main cause of source confusions and related false memories. It has been shown that the hippocampus contributes to the reconstruction processes. The hippocampus is thought be involved in relational processing, meaning the integration and recombination of information from a variety of sources (e.g., [1,103-107]).

In summary, previous studies have begun to identify the neural correlates of true and false memories at retrieval. The findings, however, are heterogeneous. Some studies indicate that the hippocampus and other regions are equally engaged by retrieval of true and false information (e.g., [102,108]), while others suggest that the regions are engaged more by retrieval of true than false memories (e.g., [95,109]), or even more by imaginary than retrieval of true memories, as in the research on future event simulation (e.g., [1,106]). Furthermore, previous evidence suggests that frontal areas are more active when more monitoring is required (e.g., source vs. old/new recognition tests; see [11] for a review). It has been shown that the hippocampus contributes to constructive memory by flexibly binding elements together in memory, sometimes resulting in false memory recognition through erroneous recombination (e.g., [1,95,109]).

## Conclusion

Within this review specific examples about the formation of false memories were given to illustrate the variety of memory errors related to diverse stages of processing. Different neuro-cognitive processes have been linked to the formation of false memories.

At encoding, processes like imagery (e.g., [28]), self-referential encoding (e.g., [12]), spreading activation or the construction of a gist trace (e.g., [35]) can lead to the formation of false memories. Data from neuroimaging studies suggest that perception (e.g., predominantly in occipital and parietal brain structures [28]) and storage processes (e.g., predominantly within the MTL [12,35]) are especially relevant for the creation of a false memory representation during encoding. The medial (e.g., self-referential processes) and lateral frontal cortex (e.g., executive functions) seem to play a key role in influencing those processes usually involved in the formation of true memories.

At the consolidation stage, false memories can be created especially due to sleep and misleading post-event information. Memory updating processes, related to the processing of recall cues together with new information probably lead to false memories due to post-event information. The imaging data suggest an involvement of perceptual brain regions [80], the MTL [78] and the PFC [78] in those memory updating processes relevant for the creation of false memories. The effect of sleep in the creation of false memories is assumed to be related to an active reorganization and integration of memories within pre-existing representations, which can result in a memory representation that can differ from what was originally encoded [60,70,71]. Semantic generalization processes in particular had been assumed to be involved in those consolidation processes leading to false memories [60]. However, imaging data indicate that sleep does not selectively enhance false memories but rather tends to promote general consolidation in hippocampo-neocortical circuits of memories subsequently associated with both true and false recollections [62].

Finally, at the retrieval stage, some studies indicate that the hippocampus and other regions are engaged equally in retrieval of true and false information (e.g., [102,108]), while others suggest that the regions are engaged more in retrieval of true than false memories (e.g., [95,109]), or even more by imaginary than retrieval of true memories (e.g., [1,106]). The hippocampus, in particular, is assumed to contribute to constructive memory, which sometimes results in false memories through incorrect recombination (e.g., [1,95,109]). Furthermore, the executive processes related to activation of prefrontal areas seems to be involved in monitoring processes, which are assumed to be important for the rejection of false memories during the recognition phase (see [11] for a review).

This overview has shown that memory formation comprises at least three different sub-processes, all of which are vulnerable for specific errors and, consequently, may result in false memories: encoding [28,30,35,78,85,99,110,111], consolidation [20,21,58-62,64,75,78] and retrieval of the learned material [2,97,102,112-115].

### Outlook

Encoding, consolidation and retrieval processes are distinguishable from each other and were divided per purpose within this overview in order to demonstrate the differences between these processing stages. However, because all processes are fully inter-connected (e.g., there is no retrieval without encoding or consolidation and success of encoding cannot be evaluated without retrieval), they ultimately have to be evaluated in relation to one another. To get a more comprehensive understanding of the formation of true and false memories, future studies should investigate the functions of each single stage of false memory formation separately by monitoring data from encoding, consolidation and retrieval phase. For example, processes specific to consolidation should not be present at encoding and processes specific to retrieval should not be present at encoding or consolidation. The manipulation of a single component at a given stage of processing by monitoring the other stages will help to identify interactions between processing stages and systems. The inclusion of implicit and explicit measures will further help to identify top-down from bottom-up processes. For example, the use of natural speech and gesture stimuli provides the possibility to define neural integration or binding processes at encoding relatively independent of subsequent memory performance [116-118]. Such complex material provides - in addition to its natural character - numerous further possibilities for manipulations of contextual (e.g., body orientation [12,119]) and content dependent factors (e.g., social vs. object related content, concrete vs. abstract utterances, related vs. unrelated speech and gesture information [12,116-120]). Measures like brain imaging (e.g., fMRI) or brain stimulation (e.g., tDCS) techniques have been found to be especially helpful in understanding relevant neuro-cognitive processes [11] and should be used more extensively and especially in combination to use advantages of each method (e.g., fMRI good spatial resolution; EEG good time resolution; direct manipulation of brain processes by means of TMS or tDCS, e.g., [121,122]). Finally, basic research should also consider the translational account of their findings for legal proceedings (e.g., interrogation), the understanding of memory distortions in psychiatric or neurological disorders, as well as treatment (e.g., cognitive behavioral therapy).

This overview about memory distortions and related brain regions represents just a very broad map of components relevant for the creation of true and false memories, which certainly does not cover all distinct sub processes and brain structures. However, this overview highlights the varieties of influences and neural structures relevant at encoding, consolidation and retrieval processes that may contribute to the formation of false memories. Other important factors with regard to the formation of false memories, such as age [101,111,123,124], current mental state or emotions [11,13,14,16-20], presence of a mental disorder [22,125], received little attention due to the limited space within this overview, but suggest further new perspectives and applications of research on memory processes. This review article is thought to motivate future studies to consider encoding, consolidation and retrieval, as well as the different related systems, in the investigation of the formation of false memories.

## Competing interests

The author declares that he has no competing interests.

## References

[B1] Addis DR Wong AT Schacter DL Remembering the past and imagining the future: common and distinct neural substrates during event construction and elaboration Neuropsychologia 2007 45 7 1363 1377 10.1016/j.neuropsychologia.2006.10.016 17126370 PMC1894691

[B2] Schacter DL Slotnick SD The cognitive neuroscience of memory distortion Neuron 2004 44 1 149 160 10.1016/j.neuron.2004.08.017 15450167

[B3] Schacter DL The seven sins of memory: how the mind forgets and remembers 2001 Houghton Mifflin, Boston

[B4] Squire LR Memory and brain systems: 1969–2009 J Neurosci 2009 29 41 12711 12716 10.1523/JNEUROSCI.3575-09.2009 19828780 PMC2791502

[B5] Squire LR Memory systems of the brain: a brief history and current perspective Neurobiol Learn Mem 2004 82 3 171 177 10.1016/j.nlm.2004.06.005 15464402

[B6] Henke K A model for memory systems based on processing modes rather than consciousness Nat Rev Neurosci 2010 11 7 523 532 10.1038/nrn2850 20531422

[B7] Tulving E Tulving E, Donaldson W Episodic and Semantic Memory Organization of memory 1972 Academic, New York 381 402

[B8] Tulving E Episodic memory: from mind to brain Annu Rev Psychol 2002 53 1 25 10.1146/annurev.psych.53.100901.135114 11752477

[B9] Shimamura AP Squire LR A neuropsychological study of fact memory and source amnesia J Exp Psychol Learn Mem Cogn 1987 13 3 464 473 2956356 10.1037//0278-7393.13.3.464

[B10] Wheeler MA Stuss DT Tulving E Toward a theory of episodic memory: the frontal lobes and autonoetic consciousness Psychol Bull 1997 121 3 331 354 9136640 10.1037/0033-2909.121.3.331

[B11] Mitchell KJ Johnson MK Source monitoring 15 years later: what have we learned from fMRI about the neural mechanisms of source memory? Psychol Bull 2009 135 4 638 677 19586165 10.1037/a0015849 PMC2859897

[B12] Straube B Green A Chatterjee A Kircher T Encoding social interactions: the neural correlates of true and false memories J Cogn Neurosci 2011 23 2 306 324 10.1162/jocn.2010.21505 20433241

[B13] Gallo DA False memories and fantastic beliefs: 15 years of the DRM illusion Mem Cognit 2010 38 7 833 848 10.3758/MC.38.7.833 20921097

[B14] Jacoby LL Wahlheim CN Rhodes MG Daniels KA Rogers CS Learning to diminish the effects of proactive interference: reducing false memory for young and older adults Mem Cognit 2010 38 6 820 829 10.3758/MC.38.6.820 PMC3030918 20852244

[B15] Long DL Prat C Johns C Morris P Jonathan E The importance of knowledge in vivid text memory: an individual-differences investigation of recollection and familiarity Psychon Bull Rev 2008 15 3 604 609 10.3758/PBR.15.3.604 18567262 PMC2674846

[B16] Brainerd CJ Reyna VF Aydin C Remembering in contradictory minds: disjunction fallacies in episodic memory J Exp Psychol Learn Mem Cogn 2010 36 3 711 735 20438268 10.1037/a0018995

[B17] Koo M Oishi S False memory and the associative network of happiness Pers Soc Psychol Bull 2009 35 2 212 220 10.1177/0146167208327191 19141625

[B18] El Sharkawy J Groth K Vetter C Beraldi A Fast K False memories of emotional and neutral words Behav Neurol 2008 19 1–2 7 11 18413909 10.1155/2008/587239 PMC5452470

[B19] Laney C Loftus EF Emotional content of true and false memories Memory 2008 16 5 500 516 10.1080/09658210802065939 18569679

[B20] Smeets T Otgaar H Candel I Wolf OT True or false? Memory is differentially affected by stress-induced cortisol elevations and sympathetic activity at consolidation and retrieval Psychoneuroendocrinology 2008 33 10 1378 1386 10.1016/j.psyneuen.2008.07.009 18790572

[B21] Baym CL Gonsalves BD Comparison of neural activity that leads to true memories, false memories, and forgetting: An fMRI study of the misinformation effect Cogn Affect Behav Neurosci 2010 10 3 339 348 10.3758/CABN.10.3.339 20805535

[B22] Bhatt R Laws KR McKenna PJ False memory in schizophrenia patients with and without delusions Psychiatry Res 2010 178 2 260 265 10.1016/j.psychres.2009.02.006 20466436

[B23] Klumpp H Amir N Garfinkel SN False memory and obsessive-compulsive symptoms Depress Anxiety 2009 26 5 396 402 10.1002/da.20526 18839404 PMC3666829

[B24] Moritz S Woodward TS Metacognitive control over false memories: a key determinant of delusional thinking Curr Psychiatry Rep 2006 8 3 184 190 10.1007/s11920-006-0022-2 19817068

[B25] Brainerd CJ Reyna VF Ceci SJ Developmental reversals in false memory: a review of data and theory Psychol Bull 2008 134 3 343 382 18444700 10.1037/0033-2909.134.3.343

[B26] Loftus EF Planting misinformation in the human mind: a 30-year investigation of the malleability of memory Learn Mem 2005 12 4 361 366 10.1101/lm.94705 16027179

[B27] Loftus E Our changeable memories: legal and practical implications Nat Rev Neurosci 2003 4 3 231 234 10.1038/nrn1054 12612635

[B28] Gonsalves B Reber PJ Gitelman DR Parrish TB Mesulam MM Paller KA Neural evidence that vivid imagining can lead to false remembering Psychol Sci 2004 15 10 655 660 10.1111/j.0956-7976.2004.00736.x 15447635

[B29] Gonsalves B Paller KA Mistaken memories: remembering events that never happened Neuroscientist 2002 8 5 391 395 10.1177/107385802236964 12374423

[B30] Gonsalves B Paller KA Neural events that underlie remembering something that never happened Nat Neurosci 2000 3 12 1316 1321 10.1038/81851 11100153

[B31] Ratcliff R McKoon G Retrieving information from memory: spreading-activation theories versus compound-cue theories Psychol Rev 1994 101 1 177 184 discussion 185–177 8121958 10.1037/0033-295x.101.1.177

[B32] Joordens S Besner D Priming effects that span an intervening unrelated word: implications for models of memory representation and retrieval J Exp Psychol Learn Mem Cogn 1992 18 3 483 491 1534350 10.1037//0278-7393.18.3.483

[B33] Balota DA Duchek JM Spreading activation in episodic memory: further evidence for age independence Q J Exp Psychol A 1989 41 4 849 876 10.1080/14640748908402396 2587800

[B34] Dell GS A spreading-activation theory of retrieval in sentence production Psychol Rev 1986 93 3 283 321 3749399

[B35] Kim H Cabeza R Differential contributions of prefrontal, medial temporal, and sensory-perceptual regions to true and false memory formation Cereb Cortex 2007 17 9 2143 2150 17110592 10.1093/cercor/bhl122

[B36] Johnson MK Hashtroudi S Lindsay DS Source Monitoring Psychol Bull 1993 114 1 3 28 8346328 10.1037/0033-2909.114.1.3

[B37] Johnson MK Raye CL Reality monitoring Psychol Rev 1981 88 1 67 85

[B38] Cabeza R Lennartson ER False memory across languages: implicit associative response vs fuzzy trace views Memory 2005 13 1 1 5 10.1080/09658210344000161 15724903

[B39] Brainerd CJ Reyna VF Fuzzy-trace theory: Dual processes in memory, reasoning, and cognitive neuroscience Adv Child Dev Behav 2001 28 41 100 11605365 10.1016/s0065-2407(02)80062-3

[B40] Reyna VF Brainerd CJ Fuzzy-trace theory and false memory: new frontiers J Exp Child Psychol 1998 71 2 194 209 10.1006/jecp.1998.2472 9843625

[B41] Brainerd CJ Reyna VF Howe ML Kingma J The development of forgetting and reminiscence Monogr Soc Res Child Dev 1990 55 3–4 1 93 discussion 94–109 2287345

[B42] Deese J On the prediction of occurrence of particular verbal intrusions in immediate recall J Exp Psychol 1959 58 5 10.1037/h0046671 13664879

[B43] Roediger HL McDermott KB Creating false memories: Remembering words not presented in lists J Exp Psychol Learn Mem Cogn 1995 21 11

[B44] McKoon G Ratcliff R Spreading activation versus compound cue accounts of priming: mediated priming revisited J Exp Psychol Learn Mem Cogn 1992 18 6 1155 1172 1447546 10.1037//0278-7393.18.6.1155

[B45] Anderson JR Retrieval of information from long-term memory Science 1983 220 4592 25 30 10.1126/science.6828877 6828877

[B46] Collins AM Loftus EF A spreading-activation theory of semantic processing 1975 82

[B47] van Kesteren MT Rijpkema M Ruiter DJ Fernández G Retrieval of associative information congruent with prior knowledge is related to increased medial prefrontal activity and connectivity J Neurosci 2010 30 47 15888 15894 10.1523/JNEUROSCI.2674-10.2010 21106827 PMC6633736

[B48] van Kesteren MT Fernández G Norris DG Hermans EJ Persistent schema-dependent hippocampal-neocortical connectivity during memory encoding and postencoding rest in humans Proc Natl Acad Sci U S A 2010 107 16 7550 7555 10.1073/pnas.0914892107 20363957 PMC2867741

[B49] Frankland PW Bontempi B The organization of recent and remote memories Nat Rev Neurosci 2005 6 2 119 130 15685217 10.1038/nrn1607

[B50] Northoff G Qin P Feinberg TE Brain imaging of the self–conceptual, anatomical and methodological issues Conscious Cogn 2011 20 1 52 63 10.1016/j.concog.2010.09.011 20932778

[B51] Northoff G Heinzel A de Greck M Bermpohl F Dobrowolny H Panksepp J Self-referential processing in our brain–a meta-analysis of imaging studies on the self Neuroimage 2006 31 1 440 457 10.1016/j.neuroimage.2005.12.002 16466680

[B52] Benoit RG Gilbert SJ Volle E Burgess PW When I think about me and simulate you: medial rostral prefrontal cortex and self-referential processes Neuroimage 2010 50 3 1340 1349 10.1016/j.neuroimage.2009.12.091 20045478

[B53] Kircher TT Brammer M Bullmore E Simmons A Bartels M David AS The neural correlates of intentional and incidental self processing Neuropsychologia 2002 40 6 683 692 10.1016/S0028-3932(01)00138-5 11792407

[B54] Kircher TT Senior C Phillips ML Benson PJ Bullmore ET Brammer M Simmons A Williams SC Bartels M David AS Towards a functional neuroanatomy of self processing: effects of faces and words Brain Res Cogn Brain Res 2000 10 1–2 133 144 10978701 10.1016/s0926-6410(00)00036-7

[B55] Northoff G Bermpohl F Cortical midline structures and the self Trends Cogn Sci 2004 8 3 102 107 10.1016/j.tics.2004.01.004 15301749

[B56] Zaragoza MS Mitchell KJ Payment K Drivdahl S False Memories for Suggestions: The Impact of Conceptual Elaboration J Mem Lang 2011 64 1 18 31 10.1016/j.jml.2010.09.004 21103451 PMC2981863

[B57] Wright DB Loftus EF How misinformation alters memories J Exp Child Psychol 1998 71 2 155 164 10.1006/jecp.1998.2467 9843620

[B58] Payne JD Schacter DL Propper RE Huang LW Wamsley EJ Tucker MA Walker MP Stickgold R The role of sleep in false memory formation Neurobiol Learn Mem 2009 92 3 327 334 10.1016/j.nlm.2009.03.007 19348959 PMC2789473

[B59] Fenn KM Gallo DA Margoliash D Roediger HL Nusbaum HC Reduced false memory after sleep Learn Mem 2009 16 9 509 513 10.1101/lm.1500808 19706833

[B60] Diekelmann S Born J Wagner U Sleep enhances false memories depending on general memory performance Behav Brain Res 2010 208 2 425 429 10.1016/j.bbr.2009.12.021 20035789

[B61] Diekelmann S Landolt HP Lahl O Born J Wagner U Sleep loss produces false memories PLoS One 2008 3 10 e3512 10.1371/journal.pone.0003512 18946511 PMC2567433

[B62] Darsaud A Dehon H Lahl O Sterpenich V Boly M Dang-Vu T Desseilles M Gais S Matarazzo L Peters F Does sleep promote false memories? J Cogn Neurosci 2011 23 1 26 40 10.1162/jocn.2010.21448 20146605

[B63] Stickgold R Sleep-dependent memory consolidation Nature 2005 437 7063 1272 1278 10.1038/nature04286 16251952

[B64] Diekelmann S Born J The memory function of sleep Nat Rev Neurosci 2010 11 2 114 126 20046194 10.1038/nrn2762

[B65] Stickgold R Walker MP Memory consolidation and reconsolidation: what is the role of sleep? Trends Neurosci 2005 28 8 408 415 10.1016/j.tins.2005.06.004 15979164

[B66] Tamminen J Payne JD Stickgold R Wamsley EJ Gaskell MG Sleep spindle activity is associated with the integration of new memories and existing knowledge J Neurosci 2010 30 43 14356 14360 10.1523/JNEUROSCI.3028-10.2010 20980591 PMC2989532

[B67] Ellenbogen JM Payne JD Stickgold R The role of sleep in declarative memory consolidation: passive, permissive, active or none? Curr Opin Neurobiol 2006 16 6 716 722 10.1016/j.conb.2006.10.006 17085038

[B68] Ellenbogen JM Hulbert JC Stickgold R Dinges DF Thompson-Schill SL Interfering with theories of sleep and memory: sleep, declarative memory, and associative interference Curr Biol 2006 16 13 1290 1294 10.1016/j.cub.2006.05.024 16824917

[B69] Diekelmann S Born J One memory, two ways to consolidate? Nat Neurosci 2007 10 9 1085 1086 10.1038/nn0907-1085 17726473

[B70] Fenn KM Nusbaum HC Margoliash D Consolidation during sleep of perceptual learning of spoken language Nature 2003 425 6958 614 616 10.1038/nature01951 14534586

[B71] Wagner U Gais S Haider H Verleger R Born J Sleep inspires insight Nature 2004 427 6972 352 355 10.1038/nature02223 14737168

[B72] Kuriyama K Stickgold R Walker MP Sleep-dependent learning and motor-skill complexity Learn Mem 2004 11 6 705 713 10.1101/lm.76304 15576888 PMC534699

[B73] Drosopoulos S Schulze C Fischer S Born J Sleep’s function in the spontaneous recovery and consolidation of memories J Exp Psychol Gen 2007 136 2 169 183 17500644 10.1037/0096-3445.136.2.169

[B74] Loftus EF Palmer JC Reconstruction of auto-mobile destruction: An example of the interaction between language and memory Journal of Verbal Learning and Verbal Behaviour 1974 13 5

[B75] Ecker UK Lewandowsky S Swire B Chang D Correcting false information in memory: manipulating the strength of misinformation encoding and its retraction Psychon Bull Rev 2011 18 3 570 578 10.3758/s13423-011-0065-1 21359617

[B76] Zhu B Chen C Loftus EF Lin C He Q Li H Xue G Lu Z Dong Q Individual differences in false memory from misinformation: cognitive factors Memory 2010 18 5 543 555 10.1080/09658211.2010.487051 20623420

[B77] Loftus EF Searching for the neurobiology of the misinformation effect Learn Mem 2005 12 1 1 2 10.1101/lm.90805 15687226

[B78] Okado Y Stark CE Neural activity during encoding predicts false memories created by misinformation Learn Mem 2005 12 1 3 11 10.1101/lm.87605 15687227 PMC548489

[B79] Loftus EF Hoffman HG Misinformation and memory: the creation of new memories J Exp Psychol Gen 1989 118 1 100 104 2522502 10.1037//0096-3445.118.1.100

[B80] Stark CE Okado Y Loftus EF Imaging the reconstruction of true and false memories using sensory reactivation and the misinformation paradigms Learn Mem 2010 17 10 485 488 10.1101/lm.1845710 20861170

[B81] Slotnick SD Schacter DL The nature of memory related activity in early visual areas Neuropsychologia 2006 44 14 2874 2886 10.1016/j.neuropsychologia.2006.06.021 16901520

[B82] Slotnick SD Schacter DL A sensory signature that distinguishes true from false memories Nat Neurosci 2004 7 6 664 672 10.1038/nn1252 15156146

[B83] Slotnick SD Visual memory and visual perception recruit common neural substrates Behav Cogn Neurosci Rev 2004 3 4 207 221 10.1177/1534582304274070 15812107

[B84] Watson JM McDermott KB Balota DA Attempting to avoid false memories in the Deese/Roediger-McDermott paradigm: assessing the combined influence of practice and warnings in young and old adults Mem Cognit 2004 32 1 135 141 10.3758/BF03195826 15078050

[B85] Sederberg PB Schulze-Bonhage A Madsen JR Bromfield EB Litt B Brandt A Kahana MJ Gamma oscillations distinguish true from false memories Psychol Sci 2007 18 11 927 932 10.1111/j.1467-9280.2007.02003.x 17958703 PMC2897900

[B86] Seamon JG Luo CR Shulman EP Toner SK Caglar S False memories are hard to inhibit: differential effects of directed forgetting on accurate and false recall in the DRM procedure Memory 2002 10 4 225 237 10.1080/09658210143000344 12097208

[B87] Weinstein Y Shanks DR Rapid induction of false memory for pictures Memory 2010 18 5 533 542 10.1080/09658211.2010.483232 20623419

[B88] Turner MS Cipolotti L Shallice T Spontaneous confabulation, temporal context confusion and reality monitoring: a study of three patients with anterior communicating artery aneurysms J Int Neuropsychol Soc 2010 16 6 984 994 10.1017/S1355617710001104 20961471

[B89] Schacter DL Guerin SA St Jacques PL Memory distortion: an adaptive perspective Trends Cogn Sci 2011 15 10 467 474 10.1016/j.tics.2011.08.004 21908231 PMC3183109

[B90] Deese J On the prediction of occurrence of particular verbal intrusions in immediate recall Journal of Experimental Psychology 1959 58 5 10.1037/h0046671 13664879

[B91] Roediger HL Watson JM McDermott KB Gallo DA Factors that determine false recall: a multiple regression analysis Psychon Bull Rev 2001 8 3 385 407 10.3758/BF03196177 11700893

[B92] McDonough IM Gallo DA Autobiographical elaboration reduces memory distortion: cognitive operations and the distinctiveness heuristic J Exp Psychol Learn Mem Cogn 2008 34 6 1430 1445 18980406 10.1037/a0013013

[B93] Harrison Y Horne JA The impact of sleep deprivation on decision making: a review J Exp Psychol Appl 2000 6 3 236 249 11014055 10.1037//1076-898x.6.3.236

[B94] Schacter DL Reiman E Curran T Yun LS Bandy D McDermott KB Roediger HL Neuroanatomical correlates of veridical and illusory recognition memory: evidence from positron emission tomography Neuron 1996 17 2 267 274 10.1016/S0896-6273(00)80158-0 8780650

[B95] Cabeza R Rao SM Wagner AD Mayer AR Schacter DL Can medial temporal lobe regions distinguish true from false? An event-related functional MRI study of veridical and illusory recognition memory Proc Natl Acad Sci U S A 2001 98 8 4805 4810 10.1073/pnas.081082698 11287664 PMC31915

[B96] Schacter DL Illusory memories: a cognitive neuroscience analysis Proc Natl Acad Sci U S A 1996 93 24 13527 13533 10.1073/pnas.93.24.13527 8942967 PMC33641

[B97] Curran T Schacter DL Norman KA Galluccio L False recognition after a right frontal lobe infarction: Memory for general and specific information Neuropsychologia 1997 35 7 1035 1049 10.1016/S0028-3932(97)00029-8 9226663

[B98] Ciaramelli E Ghetti S Frattarelli M Làdavas E When true memory availability promotes false memory: evidence from confabulating patients Neuropsychologia 2006 44 10 1866 1877 10.1016/j.neuropsychologia.2006.02.008 16580028

[B99] Schacter DL Norman KA Koutstaal W The cognitive neuroscience of constructive memory Annu Rev Psychol 1998 49 289 318 10.1146/annurev.psych.49.1.289 9496626

[B100] Abe N Okuda J Suzuki M Sasaki H Matsuda T Mori E Tsukada M Fujii T Neural correlates of true memory, false memory, and deception Cereb Cortex 2008 18 12 2811 2819 10.1093/cercor/bhn037 18372290 PMC2583150

[B101] Dennis NA Kim H Cabeza R Age-related differences in brain activity during true and false memory retrieval J Cogn Neurosci 2008 20 8 1390 1402 10.1162/jocn.2008.20096 18303982 PMC3114875

[B102] Garoff-Eaton RJ Slotnick SD Schacter DL Not all false memories are created equal: the neural basis of false recognition Cereb Cortex 2006 16 11 1645 1652 16400158 10.1093/cercor/bhj101

[B103] Addis DR Schacter DL Constructive episodic simulation: temporal distance and detail of past and future events modulate hippocampal engagement Hippocampus 2008 18 2 227 237 10.1002/hipo.20405 18157862

[B104] Schacter DL Addis DR On the nature of medial temporal lobe contributions to the constructive simulation of future events Philos Trans R Soc Lond B Biol Sci 2009 364 1521 1245 1253 10.1098/rstb.2008.0308 19528005 PMC2666708

[B105] Schacter DL Addis DR Buckner RL Episodic simulation of future events: concepts, data, and applications Ann N Y Acad Sci 2008 1124 39 60 10.1196/annals.1440.001 18400923

[B106] Schacter DL Addis DR Constructive memory: the ghosts of past and future Nature 2007 445 7123 27 10.1038/445027a 17203045

[B107] Schacter DL Addis DR The cognitive neuroscience of constructive memory: remembering the past and imagining the future Philos Trans R Soc Lond B Biol Sci 2007 362 1481 773 786 10.1098/rstb.2007.2087 17395575 PMC2429996

[B108] Schacter DL Buckner RL Koutstaal W Dale AM Rosen BR Late onset of anterior prefrontal activity during true and false recognition: an event-related fMRI study Neuroimage 1997 6 4 259 269 10.1006/nimg.1997.0305 9417969

[B109] Giovanello KS Kensinger EA Wong AT Schacter DL Age-related neural changes during memory conjunction errors J Cogn Neurosci 2010 22 7 1348 1361 10.1162/jocn.2009.21274 19445606 PMC3852424

[B110] Chen JC Li W Westerberg CE Tzeng OJ Test-item sequence affects false memory formation: an event-related potential study Neurosci Lett 2008 431 1 51 56 10.1016/j.neulet.2007.11.020 18155357

[B111] Dennis NA Kim H Cabeza R Effects of aging on true and false memory formation: an fMRI study Neuropsychologia 2007 45 14 3157 3166 10.1016/j.neuropsychologia.2007.07.003 17716696

[B112] Hanczakowski M Mazzoni G Both differences in encoding processes and monitoring at retrieval reduce false alarms when distinctive information is studied Memory 2011 19 3 280 289 10.1080/09658211.2011.558514 21500088

[B113] Garoff-Eaton RJ Kensinger EA Schacter DL The neural correlates of conceptual and perceptual false recognition Learn Mem 2007 14 10 684 692 10.1101/lm.695707 17911372 PMC2044559

[B114] Dodson CS Hege AC Speeded retrieval abolishes the false-memory suppression effect: evidence for the distinctiveness heuristic Psychon Bull Rev 2005 12 4 726 731 10.3758/BF03196764 16447388

[B115] Arndt J Reder LM The effect of distinctive visual information on false recognition J Mem Lang 2003 48 1 1 15 10.1016/S0749-596X(02)00518-119079562

[B116] Straube B Green A Bromberger B Kircher T The differentiation of iconic and metaphoric gestures: Common and unique integration processes Hum Brain Mapp 2011 32 4 520 533 10.1002/hbm.21041 21391245 PMC6870426

[B117] Green A Straube B Weis S Jansen A Willmes K Konrad K Kircher T Neural integration of iconic and unrelated coverbal gestures: a functional MRI study Hum Brain Mapp 2009 30 10 3309 3324 10.1002/hbm.20753 19350562 PMC6870774

[B118] Kircher T Straube B Leube D Weis S Sachs O Willmes K Konrad K Green A Neural interaction of speech and gesture: differential activations of metaphoric co-verbal gestures Neuropsychologia 2009 47 1 169 179 10.1016/j.neuropsychologia.2008.08.009 18771673

[B119] Straube B Green A Jansen A Chatterjee A Kircher T Social cues, mentalizing and the neural processing of speech accompanied by gestures Neuropsychologia 2010 48 2 382 393 10.1016/j.neuropsychologia.2009.09.025 19782696

[B120] Straube B Green A Weis S Chatterjee A Kircher T Memory effects of speech and gesture binding: cortical and hippocampal activation in relation to subsequent memory performance J Cogn Neurosci 2009 21 4 821 836 10.1162/jocn.2009.21053 18578601

[B121] Boggio PS Fregni F Valasek C Ellwood S Chi R Gallate J Pascual-Leone A Snyder A Temporal lobe cortical electrical stimulation during the encoding and retrieval phase reduces false memories PLoS One 2009 4 3 e4959 10.1371/journal.pone.0004959 19319182 PMC2655647

[B122] Straube B Wolk D Chatterjee A The role of the right parietal lobe in the perception of causality: a tDCS study Exp Brain Res 2011 215 3–4 315 325 21997332 10.1007/s00221-011-2899-1

[B123] Fazio LK Marsh EJ Older, not younger, children learn more false facts from stories Cognition 2008 106 2 1081 1089 10.1016/j.cognition.2007.04.012 17540354

[B124] Lövdén M Wahlin A The sensory-cognition association in adulthood: Different magnitudes for processing speed, inhibition, episodic memory, and false memory? Scand J Psychol 2005 46 3 253 262 10.1111/j.1467-9450.2005.00455.x 15842416

[B125] Mammarella N Altamura M Padalino FA Petito A Fairfield B Bellomo A False memories in schizophrenia? An imagination inflation study Psychiatry Res 2010 179 3 267 273 10.1016/j.psychres.2009.05.005 20488556

